# Controllable Synthesis of Three-Dimensional β-NiS Nanostructured Assembly for Hybrid-Type Asymmetric Supercapacitors

**DOI:** 10.3390/nano10030487

**Published:** 2020-03-08

**Authors:** Yao Zhang, Jia Zhang, Daqian Ding, Yanfang Gao

**Affiliations:** Xincheng District, No. 49 Aimin Street, Inner Mongolia University of Technology, College of Chemical Engineering, Hohhot 010051, China; zhangyao201010@163.com (Y.Z.);

**Keywords:** nanostructured β-NiS, controllable synthesis, hybrid-type asymmetric supercapacitors

## Abstract

The process of energy storage in supercapacitors via the surface reaction of the electrode can lead to a significant perfecting of the electrochemical performance of supercapacitors, for developing the morphologies of materials and increasing the specific surface areas of the electrodes. Three-dimensional nickel sulfide (NiS) superstructures with nanomorphologies, viz. coral-like, urchin-like, flake-like, and flower-like, are synthesized through simple solvothermal methods, without any template. The nanostructured flower-like β-NiS demonstrates, not only a remarkable specific capacitance of 2425.89 F·g^−1^ at the current densities of 1 A·g^−1^, but also an excellent cycling stability of approximately 100% (at the current density of 10 A·g^−1^ over 5000 cycles). Moreover, the hybrid-type asymmetric supercapacitor, constructed from the flower-like β-NiS positive electrode and active carbon negative electrode, exhibits an energy density of 42.12 Wh·kg^−1^ at a power density of 28.8 kW·kg^−1^.

## 1. Introduction

The general applications of the renewable energy, the rapid development of the electric vehicle industry, and the constructions of smart grids paved the way for the energy storage technology to become the key link in the process of energy development [1,2,3]. In the field of electrochemical energy storage, the electrochemical performances of hybrid-type asymmetric supercapacitors are expected to catch up with those of traditional chemical power sources, due to their high-power delivery and their long durability [4,5,6]. At present, the researches have focused on promoting the energy density of supercapacitors, while maintaining their power density. The electrode materials and the potential window of supercapacitors are the decisive factors in enhancing the energy density of supercapacitors [7,8]. Hence, designing and developing high specific capacitance electrode materials and assembling the electrode materials into asymmetric supercapacitors are the keys to the development of high energy density asymmetric supercapacitor devices.

Transition metal compounds, which store charges by reversible redox reactions, usually present a higher specific capacitance than electrochemical double-layer capacitance materials [9,10,11]. Among transition metal compounds, nickel-based materials have received much attention from researchers since they became known for enabling favorable electrochemical performances [12,13,14,15]. In particular, nickel sulfide has stood out not only because of its high theoretical specific capacitance, rapid redox rate, and excellent electronic conductivity, but also its diverse morphologies and multiple chemical states during the electrochemical process [16,17,18,19,20]. Recently, the rose-like Ni_3_S_4_ electrodes were successfully synthesized, and they exhibited a specific capacitance of 1797.5 F·g^−1^ (0.5 A·g^−1^) [21]. In addition, the double-shelled hollow nickel sulfides were prepared by using different a-Fe_2_O_3_ templates, and the asymmetrical supercapacitor, which used the nickel sulfides as an anode, presented a high energy density of 43.7 Wh·kg^−1^ at the power density of 664 W·kg^−1^ [22]. Moreover, Huh and Han et al. [23] performed a rational design of the forest-like nickel sulfide hierarchical architectures, and discussed the electrochemical performance of the forest-like nickel sulfide. The multiple-phase nickel sulfide was otherwise designed and synthesized by some investigators [24,25,26]. Notably, in almost all the cases discussed above, the nickel sulfide obtained for the supercapacitor exhibited favorable energy storage properties. However, there is no systematic discussion regarding the nanostructure and the electrochemical performance of nickel sulfide. Hence, it is imperative to promote the electrochemical performances of nickel sulfide materials for supercapacitor applications, which consist of various nanostructured morphologies, surface areas, and specific capacitance properties, in addition to chemical synthesis mechanisms.

In this study, a diverse morphological β-nickel sulfide (β-NiS) was synthesized using a two-step solvothermal method that involved the tuning of the solvent volume ratios of ethanol, deionized water, and glycol. The highest specific surface area of the flower-like β-NiS compound could reach up to 411.2 m^2^·g^−1^. The flower-like β-NiS electrode presented excellent electrochemical performances, for which the specific capacitance was 2424.89 F·g^−1^ (1 A·g^−1^), and the retention rate of capacitance was approximately 100% (over 5000 cycles). In addition, the flower-like β-NiS//AC asymmetrical device achieved a high energy density of 42.12 Wh·kg^−1^.

## 2. Materials and Methods

### 2.1. Materials

All the reagents for this study were used as received without further purification. Table S1 shows the physical features of the deionized water, ethanol, and glycol [27].

### 2.2. Preparation of Nickel Sulfide Precursors

The different morphological NiS materials were synthesized by a two-step solvothermal method, which consisted in dissolving 3.0 mmol of Ni(NO_3_)_2_ 6H_2_O into 30 mL of solvent (the volume ratios of ethanol, deionized water, and glycol are shown in Table 1), to obtain the green solutions, under vigorous stirring. Then, 12.0 mmol of urea was added into each of the aforementioned solution. After stirring, the four resulting solutions were transferred into 50 mL Teflon-lined stainless autoclaves, and kept at 180 °C for 6 h. After the solvothermal treatment, the green precipitates were collected, washed, and vacuum-dried at 80 °C overnight.

### 2.3. Preparation of Different Morphological Nickel Sulfides

The 0.2 g proportion mentioned above involved four precursor powders, which, with 0.6 g of CH_3_CSNH_2_ (Thioacetamide, TTA), were dispersed into 20 mL of deionized water. After stirring, the suspensions were transferred into autoclaves, with magnetic stirring (500 r·min^−1^), for the solvothermal treatment at 180 °C for 12 h. After the autoclaves were cooled down, four black precipitates were filtered out and washed. In the final step, the precipitates dried at 80 °C overnight.

### 2.4. Preparation of Electrode

To fabricate working electrodes, the as-prepared coral-like, urchin-like, flake-like, and flower-like β-NiS powders were pressed in the interspaces between two pieces of cleaned Ni foam (1 × 2 cm^2^), and then pressed at 10 MPa for 60 s [28]. The active material loadings of the coral-like, urchin-like, flake-like, and flower-like β-NiS electrodes were 4.5 mg, 4.4 mg, 4.5 mg, and 4.3 mg, respectively.

### 2.5. Physical Characterizations

The phase structures of the samples were characterized by X-ray diffraction (Cu Ka radiation, =1.5406 Å) (XRD, D/MAX-2500, Rigaku). The morphologies and structures of the as-obtained samples were observed by scanning electron microscopy (SEM, Quanta 650, FEI), transmission electron microscopy, high-resolution transmission electron microscopy, selected area electron diffraction, and energy dispersive X-ray spectrometry (TEM, HRTEM, SAED and EDS, F20, Tecnai G2). The nitrogen adsorption and desorption isotherm tests for the samples were performed using a Quantachrome QuadraWinQuadraSorb SI. The specific surface areas of the samples were obtained by the Brunauer–Emmett–Teller (BET) method.

### 2.6. Electrochemical Measurements

A typical three-electrode system in a 6 mol·L^−1^ KOH solution with the as-prepared NiS, platinum mesh (2.5 × 2.5 cm^2^), and Ag/AgCl electrode as working, counter, and reference electrodes, respectively, was used for characterizing the electrochemical measurements of the different morphological NiS. The electrochemical performance of the commercially active carbon was also tested with the three-electrode system. Electrochemical impedance spectroscopy (EIS) was carried out on the Parstat 2273 (Princeton Applied Research, America), and tested in a frequency range of 100 kHz to 10 MHz, at an open circuit potential. Cyclic voltammetry (CV) and galvanostatic charge and discharge (GCD) measurements were conducted on a CHI 760E electrochemical workstation (Chenhua, Shanghai). The specific capacitance ($C_{sp}$) was expressed in the following terms:

(1) $$C_{sp}=\frac{I\Delta t}{m\Delta V}$$

where $C_{sp}$ is the specific capacitance, *I* is the discharge current, *m* is the mass of active materials, Δ*t* is the discharge time and ΔV is the potential window.

The energy density (*E*, Wh·kg^−1^) and power density (*P*, W·kg^−1^) of the NiS-based asymmetrical supercapacitors were calculated as follows:

(2) $$E=\frac{1}{2}C_{sp}V^{2}$$

(3) $$P=\frac{E}{\Delta t}$$

where $C_{sp}$ is the specific capacitance of the cell evaluated from the Equation (2), which is based on the total mass of active materials in a two-electrode system, and *V* is the cell voltage window.

## 3. Results

### 3.1. Characterizations of Different Morphological β-NiS Compounds

The crystalline structure of the synthesized β-NiS was investigated by XRD analysis. As shown in Figure S1, the diffraction peaks at 18.5°, 29.6°, 32.7°, 35.7°, 37.3°, 40.7°, 49.3°, 50.1°, and 52.1°, respectively, match well with the (110), (101), (300), (021), (220), (211), (131), (410), and (401) crystallographic planes of the rhombohedral structure of β-NiS (JCPDs card No. 12-0041, space group R3m). In addition, the lattice constants of the β-NiS were in good agreement with the theoretical lattice constants of β-NiS (a = 9.61 Å, c = 3.140 Å), which further confirmed the structure of the β-NiS compound. Furthermore, the shapes of the diffraction peaks of the synthesized β-NiS were sharp and without other impurity peaks, which confirmed that β-NiS was successfully synthesized by solvothermal methods, when the volumes of ethanol, deionized water, and glycol were 6 mL, 12 mL, and 12 mL, respectively.

**Figure 1:**
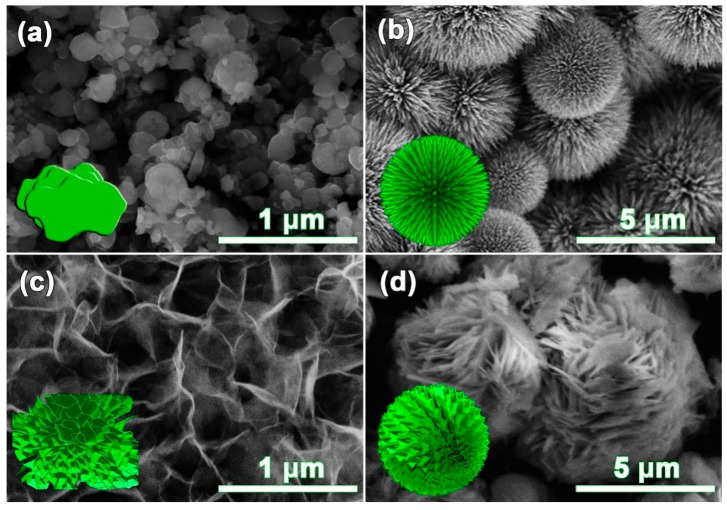
SEM images of β-NiS with different morphologies: (**a**) coral-like, (**b**) urchin-like, (**c**) flake-like and (**d**) flower-like.

The different morphological nickel sulfides were formed, which depended sensitively on the solvent. By tuning the initial volume ratios of solvents (ethanol, deionized water, and glycol), the varied structural nickel sulfides were successfully synthesized, and they were shown in Figure 1. The synthesized β-NiS showed coral-like particles with lengths of 200 to 500 nm, and a thickness of about 150 nm (Figure 1a), with only ethanol as the solvent. Figure 1b shows the urchin-like β-NiS compound, with changes in the solvent ratios of ethanol, deionized water, and glycol at 3 mL, 27 mL, and 0 mL, respectively. When the solvent ratio of ethanol, deionized water, and glycol was controlled at 12 mL, 12 mL, and 6 mL, respectively, the morphology of the β-NiS compound was transformed into the folded nanosheets (Figure 1c). By continually adjusting the ratio of ethanol, deionized water, and glycol at 6 mL, 12 mL, and 12 mL, respectively, the structure of the β-NiS compound changed into the self-assembled flower-like structure (Figure 1d).

Since the solvent physicochemical properties, such as the relative polarity index, dielectric constant, and dipole moment would greatly influence the solubility and ion transport ability of the raw materials, the solvent composition could be regarded as the morphology controller of nanostructured materials in the solvothermal process. The solvents with a different relative polarity had a different adsorption ability for each crystal face, which led to the crystal orientation growth. Generally, a solution with a low supersaturation tended to produce a low-dimensional structural material, and a solvent with a high relative polarity could be used to increase the supersaturation of the system, to obtain a high-dimensional structural material [29]. In the ethanol–deionized water–glycol solvent system, the solvent–interface interaction of glycol, with two hydroxyl groups, would be stronger than that of ethanol, containing one hydroxyl group, but weaker than that of deionized water, with the highest polarity [30]. Therefore, the aim of the controllable synthesis of the three-dimensional nanostructured β-NiS was achieved through a tuning of the volume ratios of ethanol, distilled water, and glycol, during the solvothermal process.

**Figure 2:**
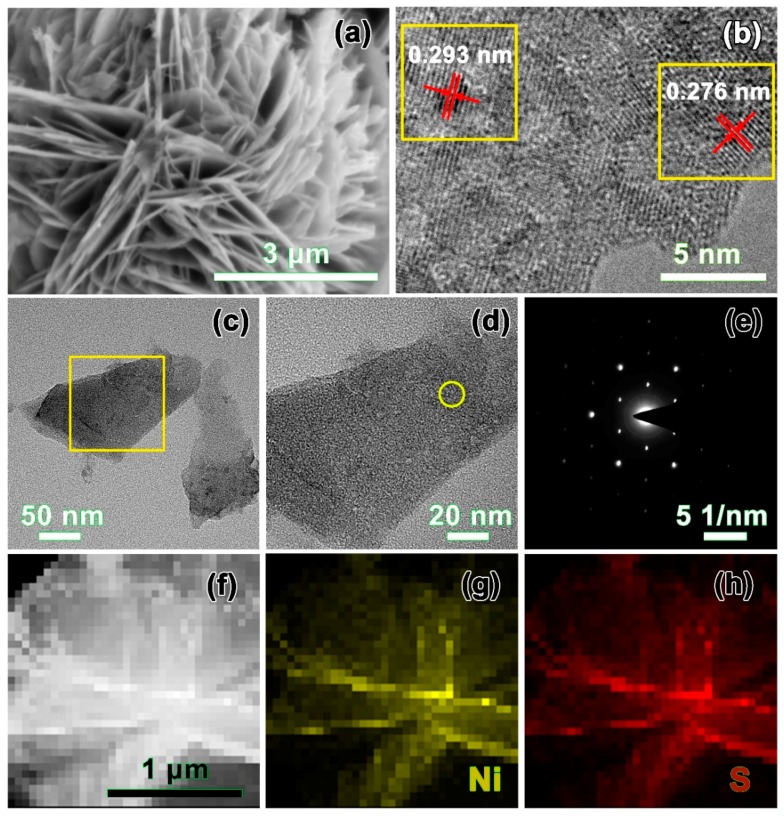
Morphological characterizations of the three-dimensional flower-like β-NiS: (**a**) enlarged SEM image, (**b**) HRTEM image, (**c**) and (**d**) TEM images, (**e**) SAED pattern, and (**f**)–(**h**) EDS mapping.

As illustrated in Figure 2a, the enlarged SEM image of the synthesized β-NiS revealed flower-like microsphere structures (about 5 μm) which were self-assembled from the nanosheets. Figure 2b shows the high-magnification HRTEM image of the flower-like β-NiS compound. The lattice interplanar spacings with interplanar distances were about 0.293 nm and 0.276 nm, corresponding to the (101) and (300) planes of the rhombohedral β-NiS, respectively [31]. The partially enlarged TEM (Figure 2c,d) presented that the synthesized β-NiS had a nanosheet configuration. The corresponding fast Fourier transform (FFT) pattern (Figure 2e) of the region, which was marked as a yellow shape in Figure 2d, confirmed the presence of a single crystallographic orientation. The visible and incontestable evidences that were extracted by the EDS mapping images (Figure 2f–h) demonstrated the homogeneous spatial distributions of Ni and S, in the basal plane in the β-NiS crystal lattice.

**Figure 3:**
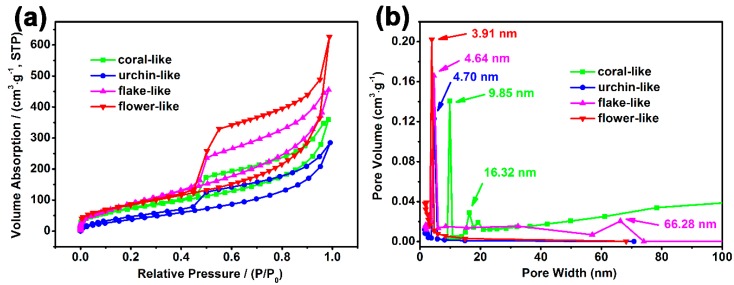
(**a**) Nitrogen adsorption–desorption isotherms and (**b**) the Barret–Joyner–Halenda (BJH) pore size distribution plots of the β-NiS with different morphologies at 77 K.

The pore structures of β-NiS with different morphologies were studied by N_2_ adsorption and desorption tests. Figure 3a shows the N_2_ adsorption and desorption isotherms of the coral-like, urchin-like, flake-like, and flower-like β-NiS compounds. All of the adsorption and desorption isotherms exhibited a IV-type with an H3 hysteresis loop, which meant that the coral-like, urchin-like, flake-like, and flower-like β-NiS compounds existed with a mesoporous structure. The specific surface areas of the coral-like, urchin-like, flake-like, and flower-like β-NiS compounds were 249.4 m^2^·g^−1^, 152.7 m^2^·g^−1^, 358.5 m^2^·g^−1^, and 411.2 m^2^·g^−1^, respectively, which were calculated using the Brunauer–Emmett–Teller (BET) method. We observed that the flower-like β-NiS compound had the largest specific surface area in four different morphological β-NiS compounds, which could increase the effective utilization of the active sites of the electrode material. Therefore, the flower-like β-NiS compound was expected to exhibit the highest electrochemical performance in the aforementioned different morphological β-NiS compounds. The Barret–Joyner–Halenda (BJH) model was used to analyze the adsorption profiles of the N_2_ adsorption and desorption curves of β-NiS with different morphologies, and the pore size distributions of the coral-like, urchin-like, flake-like, and flower-like β-NiS compounds were obtained. In Figure 3b, the pore size distributions of the coral-like, urchin-like, flake-like, and flower-like β-NiS compounds were relatively concentrated, and the pore diameters were all within the mesoporous range. However, there were macropores in the flake-like β-NiS compound, and these were ascribed to the stacking of the sheets. The pore size distribution data are shown in Table 2.

### 3.2. Electrochemical Performance of Different Morphological β-NiS Compounds

**Figure 4:**
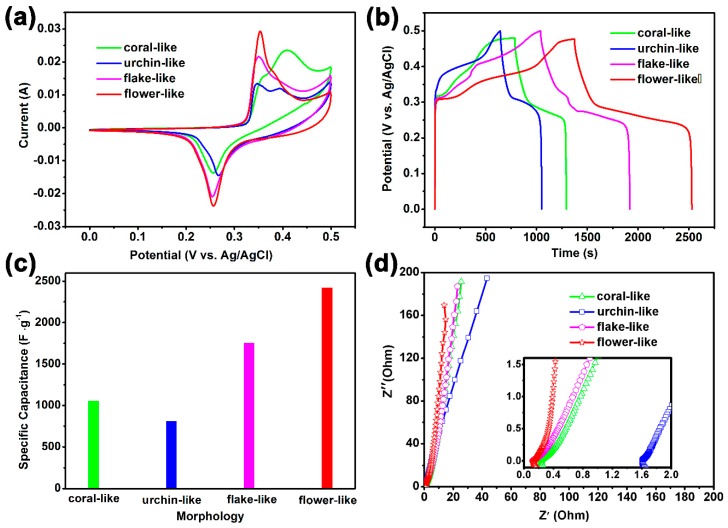
Electrochemical performances of the coral-like, urchin-like, flake-like, and flower-like β-NiS: (**a**) CV curves at a scan rate of 2 mV·s^−1^, (**b**) GCD curves at a current density of 1 A·g^−1^, (**c**) the values of the specific capacitance at a current density of 1 A·g^−1^, and (**d**) Nyquist plots and the magnified higher frequency region.

To evaluate the electrochemical performances of the as-obtained electrodes, the different morphological β-NiS compounds were characterized by CV and GCD measurements. Figure 4a shows the CV curves of β-NiS with different morphologies at a scan rate of 2 mV·s^−1^. The integral areas of the CV curve, corresponding to the flower-like β-NiS compound, was notably larger than those of the synthesized β-NiS with the flake-like, coral-like, and urchin-like morphology electrodes, which suggested a much higher capacitance. The faradaic reaction of β-NiS could be indicated as Equation (4):

(4) $$\beta -NiS+H_{2}O+OH^{-}↔\gamma -NiOOH+H_{2}S+e^{-}$$

Figure 4b presents the GCD curves of the β-NiS electrodes with different morphologies. Compared with the coral-like, urchin-like, flake-like β-NiS electrodes, the prolonged discharge time of the flower-like β-NiS electrode was suggested to have the highest specific capacitance value. Through further calculation with Equation (1), the specific capacitance of the coral-like, urchin-like, flake-like, and flower-like β-NiS electrodes were 1056.25 F·g^−1^, 814.00 F·g^−1^, 1760.00 F·g^−1^, and 2425.89 F·g^−1^ at the current density of 1 A·g^−1^, respectively, and these were consistent with the order of the integral areas of the redox peak in the CV curves of β-NiS with different morphologies in Figure 4a. As can be seen from Figure 4a,b, the energy storage performances of the supercapacitor electrodes were directly dependent on the morphology of the electrodes. Among the four different morphological β-NiS electrodes, the flower-like β-NiS electrode had the highest specific capacitance value (Figure 4c), which could be proved to be a large number of electroactive sites that participated in the redox reaction. Furthermore, the excellent electrochemical property of the flower-like β-NiS electrode was further estimated from the EIS results. Figure 4d presents the impedances of the coral-like, urchin-like, flake-like, and flower-like β-NiS electrodes. All of the Nyquist plots showed the semicircles in the high frequency region and the straight lines in low frequency region. It was obvious that the straight line in the low frequency region of the flower-like β-NiS electrode displayed the largest slope, which indicated that the flower-like β-NiS electrode had the fastest electrolyte diffusion rate among the four β-NiS compounds [32]. The results were consistent with the N_2_ adsorption and desorption tests, which indicated that the large electroactive surface could decrease the charge-transfer resistance, and improve the ion transfer rate. Figure S2 shows an equivalent circuit, including the resistance of the electrodes (R_s_), charge transfer resistance (R_ct_), diffusive resistance (W), double-layer capacitance (C_dl_), and the pseudocapacitive capacitance (C_ps_). As shown in Table S2, the fitting results of the coral-like, urchin-like, flake-like, and flower-like β-NiS electrodes were compared. The values of R_s_ and R_ct_ for the flower-like β-NiS were 0.1168 Ω and 0.2051 Ω, respectively, which indicated that the flower-like β-NiS had the lowest series resistance and charge transfer resistance among the four β-NiS electrodes.

In order to better study the electrochemical performance of the flower-like β-NiS electrode, the CV at different scanning rates and the GCD at various current density measurements were performed on the flower-like β-NiS. Figure S3 presents the slight shifts in positive and negative ways on the oxidation peaks and reduction peaks of the flower-like β-NiS with increasing scan rates, respectively, which indicates a contact deterioration between the electrode material and the electrolyte solution, at high current densities. As shown in Figure S4, all the GCD curves at different current densities of the flower-like β-NiS electrode presented the voltage plateaus, which indicated that the flower-like β-NiS was an ideal pseudocapacitive material [33]. The calculated specific capacitances of the flower-like β-NiS electrode were 2424.89, 2389.60, 1613.90, 1452.40, 1208.00, and 882.00 F·g^−1^ at the current densities of 1, 2, 5, 10, 20, and 50 A·g^−1^, respectively. The cyclic stability was also one of the important indexes for evaluating the electrochemical performance of the electrode. Figure S5 presents the 99.26% specific capacitance retention over 5000 cycles of the flower-like β-NiS electrode, which demonstrated a good electrochemical stability of the flower-like electrode. Interestingly, the increased specific capacitance after 1000 cycles could be notably observed in the life curves, which might be caused by the fact that a small amount of Ni foam reacted with the high concentration of the KOH solution at a high current density of 10 A·g^−1^, and then produced a little Ni(OH)_2_ (Figure S6) [28]. Overall, the high specific capacitance and good cycling stability of the flower-like β-NiS were attributed to the following aspects: (1) the three-dimensional flower-like β-NiS with an increased specific surface area facilitated the redox reaction and promoted the ion transport between the electrochemical active sites and the electrolyte. (2) Each nanosheet in the flower-like β-NiS was connected, which enhanced the electron transfer rate in the pseudocapacitive reaction, and reduced the charge transfer resistance of the electrode [34].

### 3.3. Asymmetric Supercapacitors

**Figure 5:**
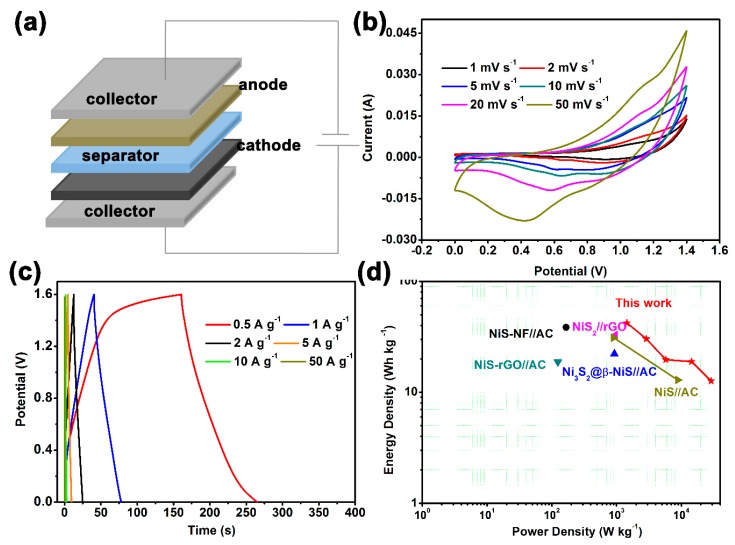
Electrochemical performances of the flower-like β-NiS//active carbon hybrid asymmetric supercapacitor: (**a**) schematic illustration of the asymmetric supercapacitor configuration, (**b**) CV curves at different scan rates, (**c**) GCD curves at various current densities, and (**d**) the Ragone plots.

As a measure for evaluating the electrochemical performance of an electrode, a two-electrode asymmetric hybrid-type supercapacitor (Figure 5a), with the β-NiS electrode as the anode, the active carbon (AC) as the cathode, and the KOH solution as the electrolyte, was assembled. The electrochemical performance of the used active carbon is shown in Figures S7 and S8. Figure 5b presents the CV curves of the β-NiS//AC supercapacitor with different scan rates in the potential window of 0–1.5 V. The shapes of the CV curves of the β-NiS//AC supercapacitor all showed redox peaks at the scan rates of 1, 2, 5, 10, 20, and 50 mV·s^−1^, which indicated that the β-NiS//AC supercapacitor showed a favorable electrochemical performance. The GCD curves of the β-NiS//AC supercapacitor are shown in Figure 5c, from which the specific capacitance values of 32.90, 23.66, 15.24, 14.75, 9.88, and 5 F·g^−1^, at various current densities (from 0.5 to 50 A·g^−1^), could be deduced. Figure 5d shows the Ragone plots of the energy density versus the power density for the β-NiS//AC supercapacitor. As calculated from the Equations (2) and (3), the energy density of β-NiS//AC was found to be over 42.12 Wh·kg^−1^ at the power density of 1440 W·kg^−1^. Even at the high power densities of 2880, 5760, 14,400, and 28,800 W·kg^−1^, the energy densities of β-NiS//AC could still reach the values of 30.28, 19.62, 18.88, and 12.64 Wh·kg^−1^, respectively. Furthermore, the electrochemical performance of the β-NiS//AC supercapacitor was relatively better than that of various transition metal sulfide//AC asymmetric supercapacitors [16,31,35,36,37]. Hence, the flower-like β-NiS compound with a special morphology and structure appeared promising for energy storage devices, which was taken into account in the prominent electrochemical performance of β-NiS.

## 4. Conclusions

In summary, β-NiS with coral-like, urchin-like, flake-like, and flower-like morphologies were successfully synthesized using a solvothermal method, and without any surfactant or templating agent. The structures and compositions of the β-NiS compounds with different morphologies were mainly characterized by the XRD, SEM, TEM, and BET measurements. The flower-like β-NiS possessed a high specific capacitance value of 2424.89 F·g^−1^ at 1 A·g^−1^. The noticeable electrochemical performance of the flower-like β-NiS benefited from its large surface area and special pore structures. The capacitance retention of the flower-like β-NiS electrode was still 99.26% after 5000 cycles at 10 A·g^−1^. For hybrid-type asymmetric supercapacitors, the β-NiS//AC exhibited a capacitance of 32.90 F·g^−1^, with a maximum energy density of 42.12 Wh·kg^−1^ and a power density of 28.8 kW·kg^−1^, which had significantly exceeded those of previously reported transition metal sulfide-based supercapacitors. Therefore, this study might provide a new way to extend the research on advancing the charge storage performance of transition metal sulfide electrodes, for highly pseudocapacitive capacitors.

## Figures and Tables

**Table 1:** Solvent volume ratios for β-NiS with coral-like, urchin-like, flake-like, and flower-like morphologies.

Materials	Volumes Ratios (mL)		
	Ethanol	Deionized Water	Glycol
coral-like β-NiS	30	0	0
urchin-like β-NiS	3	27	0
flake-like β-NiS	12	12	6
flower-like β-NiS	6	12	12

**Table 2:** Pore size distributions of β-NiS with coral-like, urchin-like, flake-like, and flower-like morphologies.

Materials	Pore Size Distribution	
	Mesopore	Macropore
coral-like β-NiS	9.85 nm and 16.32 nm	——
urchin-like β-NiS	4.70 nm	——
flake-like β-NiS	4.64 nm	66.28 nm
flower-like β-NiS	3.91 nm	——

## Supplementary Materials

The following are available online at https://www.mdpi.com/2079-4991/10/3/487/s1; Table S1: Some physical features of the used solvents. Table S2. The fitting values of R_s_, C_dl_, R_ct_, W and C_ps_ of the coral-like, urchin-like, flake-like and flower-like β-NiS electrodes through the Zsimpwin software. Figure S1: XRD pattern of the flower-like β-NiS; Figure S2: Equivalent circuit used for fitting impedance spectra; Figure S3: CV curves of the flower-like β-NiS at different scan rates of 1 mV·s^−1^, 2 mV·s^−1^, 5 mV·s^−1^, 10 mV·s^−1^, 20 mV·s^−1^, and 50 mV·s^−1^; Figure S4: GCD curves of the flower-like β-NiS at various current densities of 1 A·g^−1^, 2 A·g^−1^, 5 A·g^−1^, 10 A·g^−1^, 20 A·g^−1^, and 50 A·g^−1^; Figure S5: Cycling stability of the flower-like β-NiS at a current density of 10 A·g^−1^ for 5000 cycles; Figure S6: CV curves of the flower-like β-NiS and bare Ni foam in 6 mol·L^−1^ KOH solution at 10 A·g^−1^ at the first cycle, and (b) CV curves of the flower-like β-NiS in 6 mol·L^−1^ KOH solution at 10 A·g^−1^ after 5000 cycles, and bare Ni foam immersed into 6 mol·L^−1^ KOH solution for 8 days. Figure S7: CV curves of the active carbon at scan rates of 1 mV·s^−1^, 5 mV·s^−1^, 10 mV·s^−1^, 20 mV·s^−1^, 50 mV·s^−1^, and 100 mV·s^−1^; Figure S8: GCD curves of the active carbon at current densities of 1 A·g^−1^, 2 A·g^−1^, 5 A·g^−1^, 10 A·g^−1^, and 20 A·g^−1^.
