# Supplementary material for: Controllable Synthesis of Three-Dimensional β-NiS Nanostructured Assembly for Hybrid-Type Asymmetric Supercapacitors
Source: Nanomaterials (Basel). 2020 Mar 8;10(3):487. doi: 10.3390/nano10030487 (PMC7153257; doi:10.3390/nano10030487)
Supplement: Supplementary file 1 [file nanomaterials-10-00487-s001.pdf]

Support Information

Controllable Synthesis of Three-Dimensional β-NiS Nanostructured Assembly for Hybrid-Type Asymmetric Supercapacitors

Yao Zhang, Jia Zhang, Daqian Ding and Yanfang Gao *

Xincheng District, No. 49 Aimin Street, Inner Mongolia University of Technology, College of Chemical Engineering, Hohhot 010051, China; zhangyao201010@163.com (Y.Z.); imut_zhang@163.com (J.Z.); 13671509616@163.com (D.D.)

* Correspondence: yf_gao@imut.edu.cn.

Received: 3 February 2020; Accepted: 5 March 2020; Published: 8 March 2020

Table S1. Some physical features of the used solvents.

Solvent	deionized water	ethanol	glycol
**Formula**	H_2O	C_2H_6O	$C_2H_6O_2$
**Boiling point (℃)**	100.0	78.2	197.3
**Relative polarity index**	10.2	4.3	6.9
**Dielectric constant**	80.20	24.55	41
**Viscosity (mPa·s) (20℃)**	1.005	1.15	23
**Dipole Moment**	1.85±0.004	1.69±0.03	2.36±0.1

Table S2. The fitting values of R_s, C_{dl}, R_{ct}, W and C_{ps} of the coral-like, urchin-like, flake-like and flower-like β-NiS electrodes through the Zsimpwin software.

Electrode	R_s (Ω)	C_{dl} (F)	R_{ct} (Ω)	W (Ω)	C_{ps}(F)
corla-like β-NiS	0.2326	0.001023	0.2946	1.653	0.8364
urchin-like β-NiS	1.602	0.0008346	0.5724	1.843	0.6258
flake-like β-NiS	0.1254	0.08274	0.2735	0.7291	1.029
flower-like β-NiS	0.1168	0.08953	0.2051	0.6726	1.385

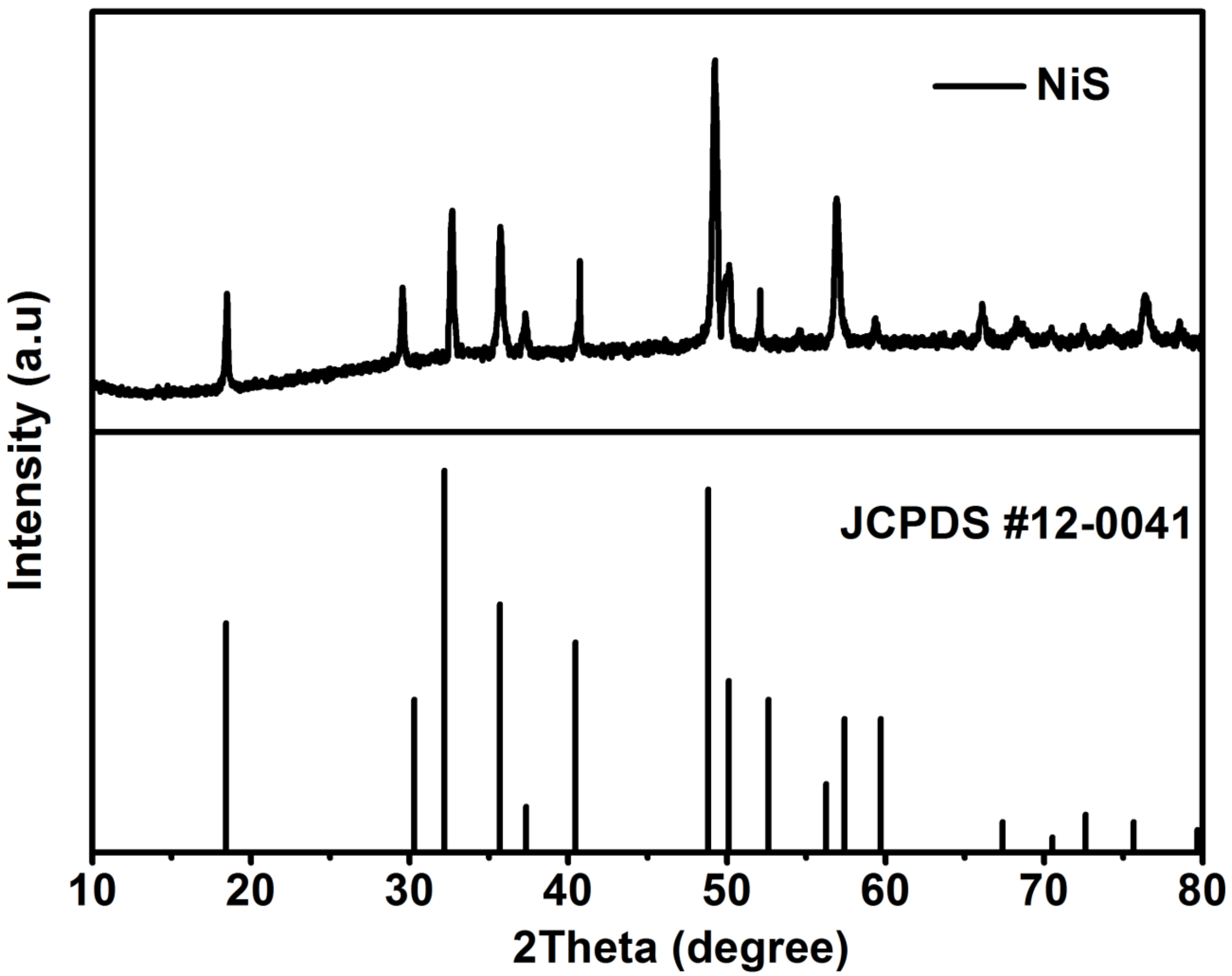

Figure S1. XRD pattern of the flower-like β-NiS.

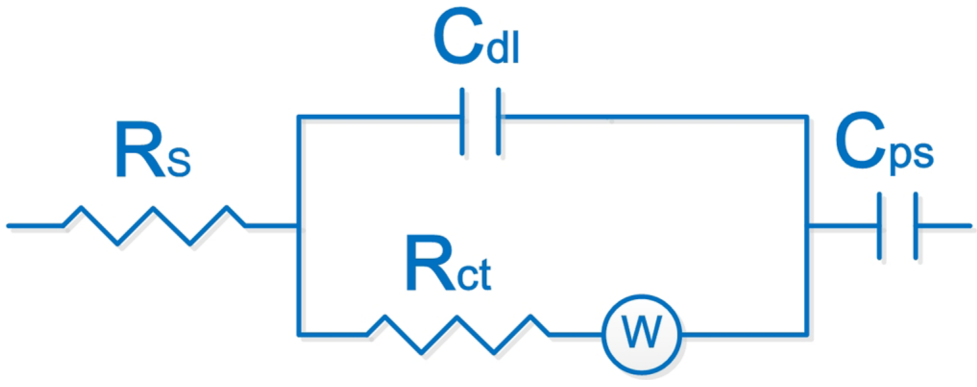

Figure S2. Equivalent circuit used for fitting impedance spectra.

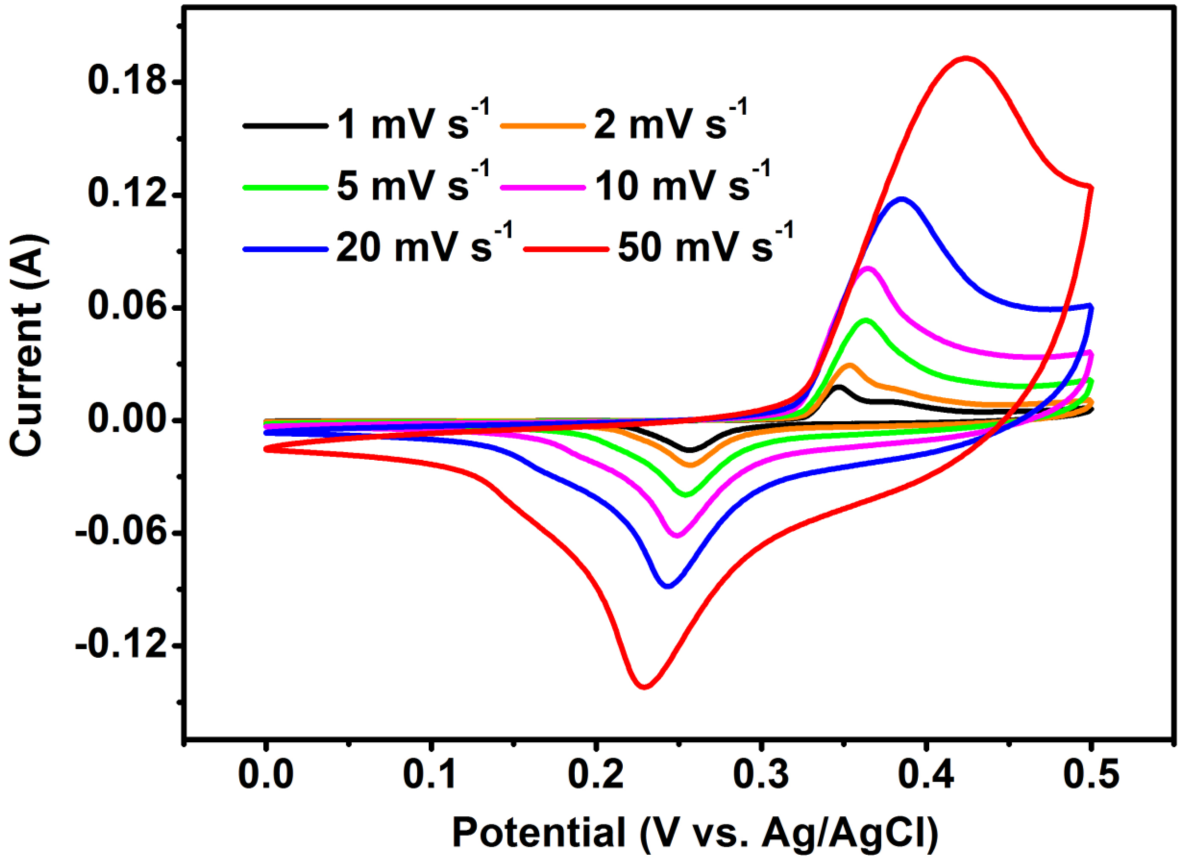

Figure S3. CV curves of the flower-like β-NiS at different scan rates of 1 mV·s^{-1}, 2 mV·s^{-1}, 5 mV·s^{-1}, 10 mV·s^{-1}, 20 mV·s^{-1} and 50 mV·s^{-1}.

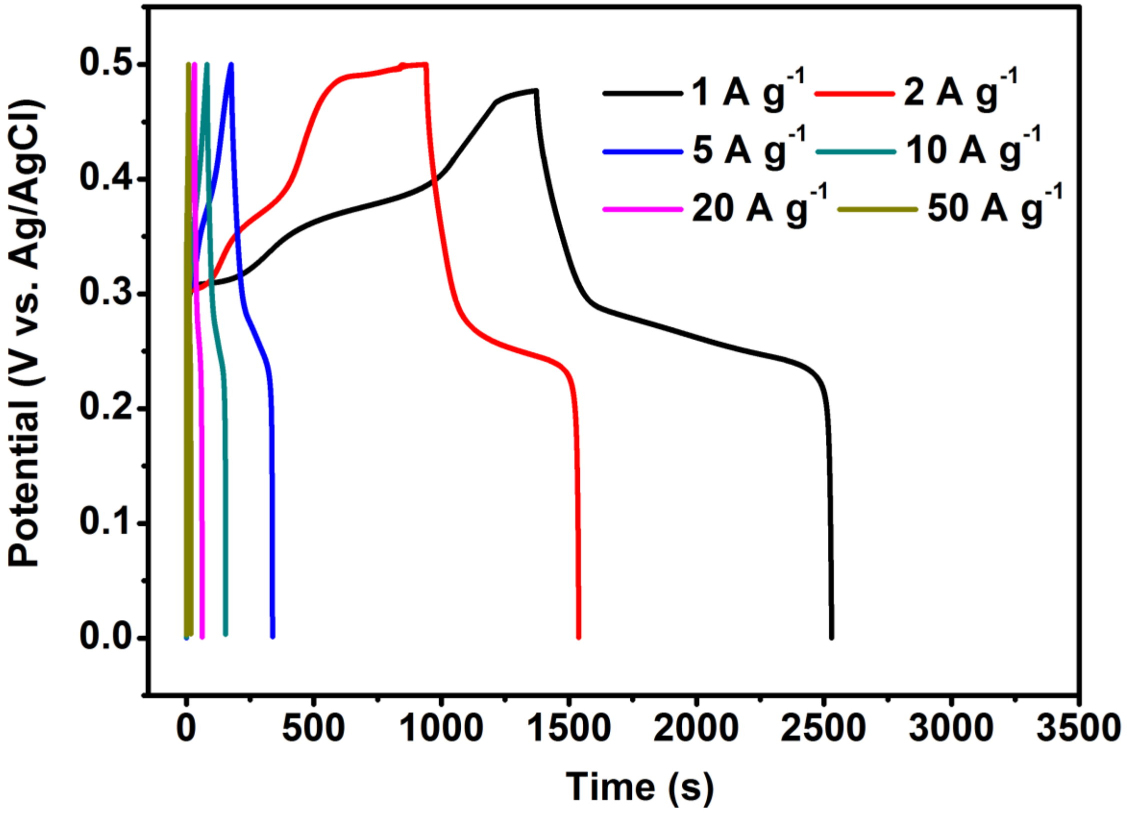

Figure S4. GCD curves of the flower-like β-NiS at various current densities of 1 A·g^{-1}, 2 A·g^{-1}, 5 A·g^{-1}, 10 A·g^{-1}, 20 A·g^{-1} and 50 A·g^{-1}.

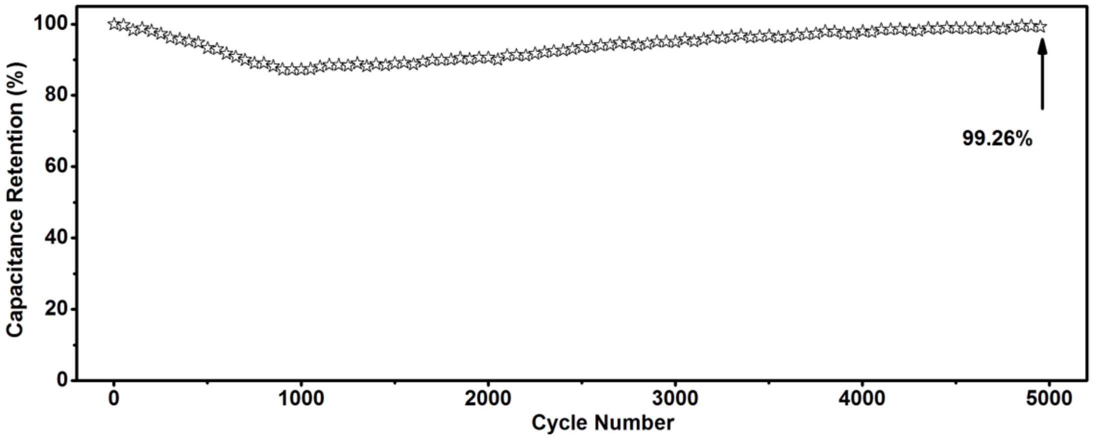

Figure S5. Cycling stability of the flower-like β-NiS at a current density of 10 A·g^{-1} for 5000 cycles.

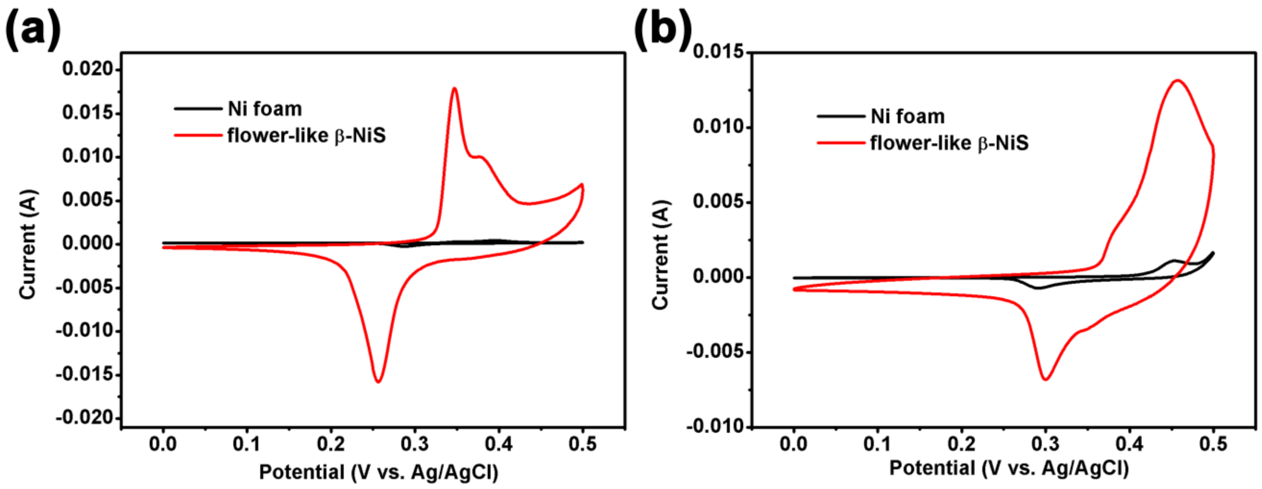

Figure S6. (a) CV curves of the flower-like β-NiS and bare Ni foam in 6 mol·L^{-1} KOH solution at 10 A·g^{-1} at the first cycle, and (b) CV curves of the flower-like β-NiS in 6 mol·L^{-1} KOH solution at 10 A·g^{-1} after 5000 cycles , and bare Ni foam immersed into 6 mol·L^{-1} KOH solution for 8 days.

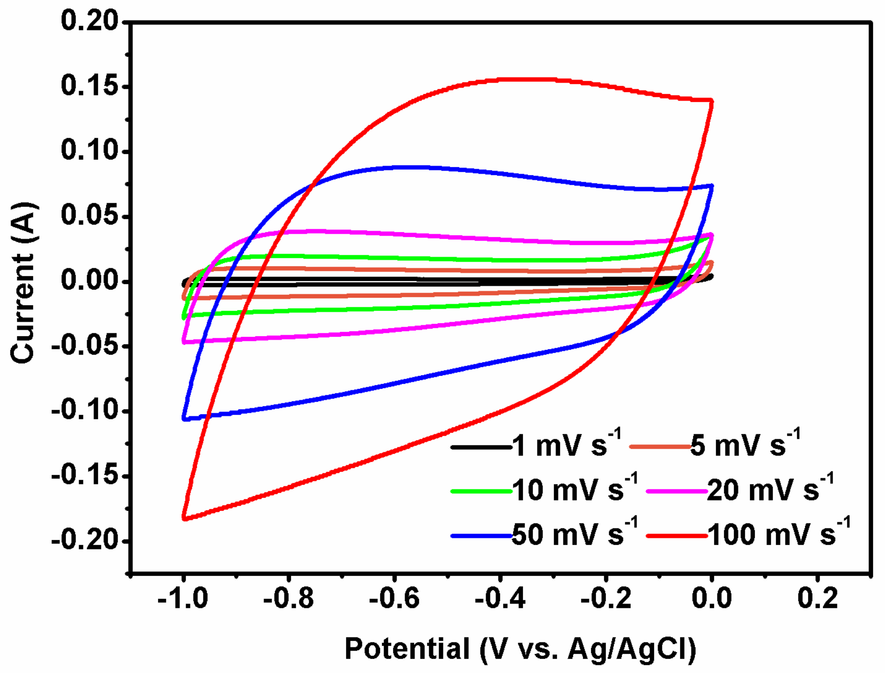

Figure S7. CV curves of the active carbon at scan rates of 1 mV·s^{-1}, 5 mV·s^{-1}, 10 mV·s^{-1}, 20 mV·s^{-1}, 50 mV·s^{-1} and 100 mV·s^{-1}.

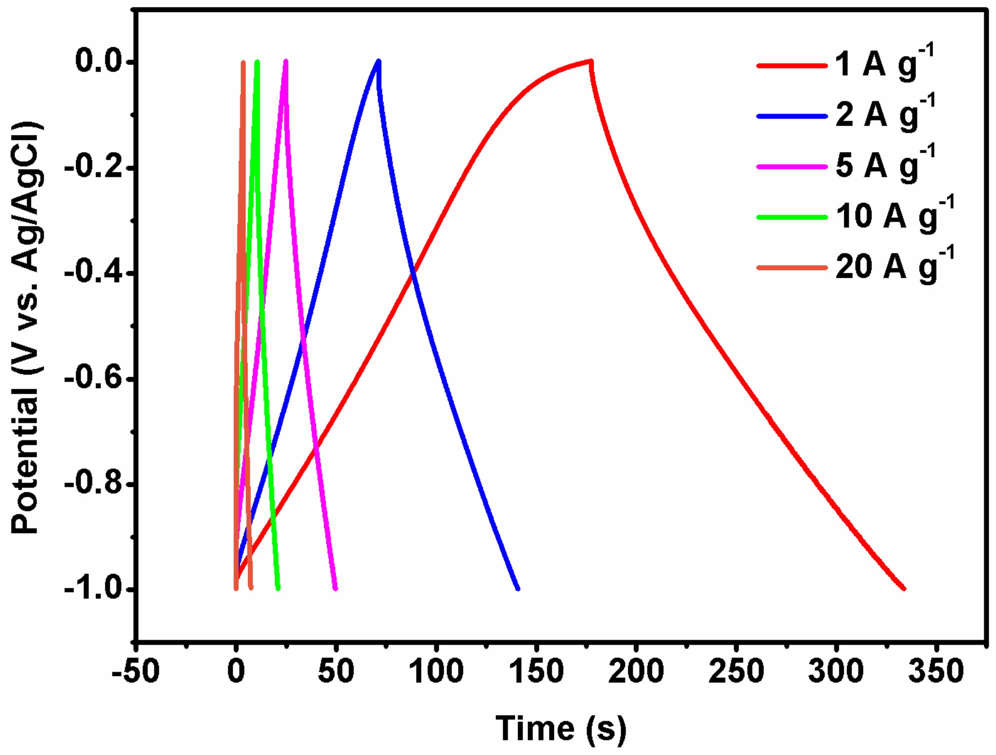

Figure S8. GCD curves of the active carbon at current densities of 1 A·g^{-1}, 2 A·g^{-1}, 5 A·g^{-1}, 10 A·g^{-1}, and 20 A·g^{-1}.
